# High-performance work practices and employee wellbeing: organizational identification as a mediator

**DOI:** 10.3389/fpsyg.2023.1175344

**Published:** 2023-07-25

**Authors:** Denise Salin, Chris Stride, Sofia Smith, Stefan Santokhie

**Affiliations:** ^1^Department of Management and Organisation, Hanken School of Economics, Helsinki, Finland; ^2^Institute of Work Psychology, SUMS, University of Sheffield, Sheffield, United Kingdom

**Keywords:** engagement, high-performance work practices, organizational identification, wellbeing, workplace bullying

## Abstract

**Aim:**

The aim of this study was to examine how high-performance work practices affect engagement and workplace bullying, two different aspects of employee wellbeing. Furthermore, the study sought to examine the potential mediating role of organizational identification in these relationships.

**Method:**

A two-wave survey study (*n* = 213) was conducted among psychologists in Finland.

**Results:**

The results showed that high-performance work practices (HPWPs) were positively associated with engagement and negatively associated with the risk of workplace bullying. Moreover, organizational identification acted as mediator of the HPWPs-engagement relationship, though alongside the significant indirect effect via organizational identification there was also a significant direct effect of HPWPs on engagement.

**Discussion:**

The study adds knowledge to ongoing debates on whether HPWPs support or undermine employee wellbeing. In particular, it extends our understanding of the association between HPWPs and relationship wellbeing, a topic that has so far received scant attention. Furthermore, the study advances our understanding of explanatory mechanisms in the HPWPs-engagement relationship and points to the importance of organizational identification for explaining why HPWPs lead to higher engagement.

## 1. Introduction

During the past decades, there has been growing interest in how human resource practices affect not only organizational performance, but also employee wellbeing (Guest, 2017; Peccei and Van De Voorde, 2019). Scholars have found that when employees experience positive attitudes and positive states of wellbeing the organization benefits from an improvement in performance (Van De Voorde et al., 2012; Ogbonnaya and Valizade, 2018). Given such performance-related benefits for the organization, it is important for organizations to have human resource practices that bolster employee wellbeing.

When studying human resource practices, the focus has typically been on high-performance work practices (HPWPs), which are defined as separate, but interconnected work and employment practices that are designed to increase employee skills and effort (Takeuchi et al., 2007). While some ambiguity remains concerning exactly which practices are to be included, there is generally widespread agreement that this group of practices includes at least sophisticated approaches to recruitment and selection, extensive investments in training, rigorous performance appraisal systems, performance-based compensation systems, and substantial employee participation and autonomy (Chuang and Liao, 2010; Boon et al., 2019).

Overall, existing research has shown strong evidence for the performance enhancing effects of such practices (Combs et al., 2006; Jiang et al., 2012). However, the effects on employee wellbeing have been more debated. In fact, there has been a long-standing debate of whether HPWPs lead to mutual gains, that is positive effects for both employer and employee, or to conflicting outcomes, that is positive effects for the employer at the expense of the employee’s health and wellbeing (Van De Voorde et al., 2012; Ogbonnaya and Messersmith, 2019). While the research evidence is inconclusive and at times contradictory, existing reviews seem to provide more support for the former (Van De Voorde et al., 2012; Peccei and Van De Voorde, 2019). Still, our understanding of the relationship between HPWPs and wellbeing remains limited and there are many open questions.

For instance, although a number of studies suggest that HPWPs have an impact on employee wellbeing, most studies have predominantly looked at certain aspects of wellbeing at the expense of others (Peccei and Van De Voorde, 2019). Wellbeing is typically defined in terms of three dimensions: happiness (e.g., job satisfaction and sense of purpose), health (e.g., stress and physical wellbeing), and relationships (social wellbeing) (Grant et al., 2007). The studies that have looked at the effect of HPWPs on wellbeing have primarily focused on aspects of happiness wellbeing and, to a lesser extent, health (see Van De Voorde et al., 2012; Peccei and Van De Voorde, 2019). Few have looked at the relationship between HPWPs and relationship (social) wellbeing, and even fewer at the underlying mechanisms and boundary conditions that drive the relationship between HPWPs and wellbeing. This study adds to existing research by studying an aspect of happiness wellbeing (i.e., engagement), as well as an aspect of relationship wellbeing (i.e., workplace bullying or the lack thereof). Furthermore, this study advances existing research by proposing organizational identification as a mechanism that explains some of the positive effects of HPWPs.

Employee engagement has been widely researched in relation to how it is affected by HPWPs. Several studies found that high performance work practices (HPWPs) have positive impacts on employee engagement (Ang et al., 2013; Van De Voorde et al., 2016; Alfes et al., 2021), which in turn has positive impacts on individual and organizational performance (Harter et al., 2002; Christian et al., 2011; Alfes et al., 2013a,b). However, little is known about mechanisms that link HPWPs and engagement. Arguments are often drawn from a social exchange perspective, suggesting that employees repay positive work experiences with positive attitudes and behaviors (Peccei and Van De Voorde, 2019). We suggest an additional alternative by drawing on social identity theory and propose that organizational identification mediates the relationship between HPWPs and employee wellbeing.

Workplace bullying has often been explained through the work environment hypothesis, which suggests that bullying largely stems from deficiencies in job design and in the work environment (Einarsen, 2000; Skogstad et al., 2011). While workplace bullying has been framed and investigated in relation to numerous organizational and job-related antecedents (Salin and Hoel, 2020), the effects of HR practices on workplace bullying has received little attention. Currently, there is ambiguity as to whether HPWPs might lead to improvements in the work environment and thereby reduce the risk of bullying (Salin and Notelaers, 2020), or whether HPWPs may rather lead to increases in incivility, workplace bullying, and abusive supervision (Samnani and Singh, 2014; Ashkanasy et al., 2016; Pichler et al., 2016). Thus, this study aims to shed more light on the nature of this contested relationship. Additionally, we draw on social identity theory and explore whether organizational identification provides an additional lens through which we can better understand the effects of HPWPs on workplace bullying.

The disputed nature of the HPWPs-relationship wellbeing association is linked to the larger discussion on whether HPWPs lead to mutual gains for organizations and employees alike, whether they lead to improved organizational performance at the expense of employee wellbeing (i.e., conflicting outcomes), or whether they in fact lead to mutual losses (Peccei and Van De Voorde, 2019). While reviews of existing research seem to rather strongly confirm a positive relationship between HPWPs and happiness wellbeing (including engagement), overall findings for health outcomes and relationship wellbeing are more mixed, and the latter so far sparsely studied (Van De Voorde et al., 2012; Peccei and Van De Voorde, 2019).

The aim of this study is to examine the impact of HPWPs on engagement and workplace bullying, two examples of employee wellbeing. The study further postulates that organizational identification mediates the relationship between HPWPs and these employee outcomes and provides an empirical examination of this. As such, the contribution of this paper is three-fold. First, it contributes to the HRM literature, furthering our understanding of how HPWPs affect happiness wellbeing and relationship wellbeing, thus contributing to the mutual gains versus conflicting outcomes debate. Second, it increases our understanding of how HPWPs affect engagement, by pointing to the role of increased organizational identification. Third, the study contributes to our understanding of workplace bullying, examining how work environment factors such as HPWPs reduce the risk of workplace bullying.

In the following section the relationship between HPWPs and employee wellbeing is discussed in more detail, thereby providing the background and theoretical argumentation for our hypotheses. Subsequently out study design, a two-wave survey among psychologists in Finland, is presented. Finally, the results of our statistical analyses are presented and the implications for research and practice discussed.

## 2. High-performance work practices and employee wellbeing

When empirically studying HRM activities, the focus has typically been on studying high-performance work practices and systems (Sun et al., 2007; Takeuchi et al., 2007; Boxall and Macky, 2009; Ogbonnaya and Valizade, 2018; Alfes et al., 2021), or highly related and overlapping concepts such as high-involvement (Wood et al., 2012) or high-commitment practices (Boon and Kalshoven, 2014; Mostafa et al., 2019). In this article we draw on findings from all three highly overlapping fields, although we ourselves use the term high-performance work practices (HPWPs). HPWPs have been defined as a group of separate but interconnected human resource (HR) practices that are effective in enhancing employees’ ability, motivation and opportunities to contribute to organizational performance and in fostering long-term competitive advantage (Combs et al., 2006; Takeuchi et al., 2007). The theoretical foundation of HPWPs largely rest on high commitment (Walton, 1985), high involvement (Lawler, 1986), and principles of management that create opportunities for employees to exchange ideas, develop their job skills, and use their knowledge to improve the organization (Wood et al., 2012).

A key aspect of HPWPs is the idea of HRM bundling (Macduffie, 1995), suggesting that to have greater impact on outcomes, it is important to ensure that HR practices fit together, thereby supporting one another (Delery, 1998). In other words, it is important to implement and study bundles of practices rather than single practices *per se*. The AMO-framework highlights that organizations need to implement HR practices that foster the Ability, Motivation, and Opportunity to participate in and contribute to organizational performance (Appelbaum et al., 2000; Boxall and Purcell, 2003). Ability-enhancing practices include, for instance, rigorous recruitment and selection procedures and extensive training; motivation-enhancing practices include, for instance, performance appraisal and compensation; opportunity-enhancing practices include, for instance, participation, empowerment, and flexible job designs (Jiang et al., 2012).

High-performance work practices have typically been conceptualized at the organizational level (Ogbonnaya and Valizade, 2018). However, reviews have found that studies are increasingly making use of employee perceptions of HPWPs rather than management-rated HPWPs (Beijer et al., 2021; van Beurden et al., 2021). Reasons for this are, for instance, that there may be differences between intended and implemented practices, that practices may be implemented differently across employees, and that employees may differ in their interpretations of these practices (*cf.* Nishii et al., 2008; Nishii and Wright, 2008). In line with this, studies have found that employees’ perceptions of HPWPs mediate the relationship between management-rated intended practices and outcomes (Elorza et al., 2016). Therefore, in this study we rely on employee perceptions of HPWPs.

An ongoing debate in the HPWPs literature is whether HPWPs lead to mutual gains for organizations and employees, whether they have conflicting outcomes (i.e., organizational performance at the expense of employee wellbeing) or whether they potentially lead to mutual losses (Peccei and Van De Voorde, 2019). To complicate matters further, empirical findings suggest effects on employee wellbeing may differ depending what aspects of wellbeing – happiness, health or relationship wellbeing – one focuses on (Van De Voorde et al., 2012; Guerci et al., 2019; Peccei and Van De Voorde, 2019). In response to this, this study seeks to examine the effect of HPWPs on two different forms of employee wellbeing, that is, engagement and workplace bullying, the former an example of happiness wellbeing and the latter an example of relationship (or social) wellbeing.

### 2.1. High-performance work practices and employee engagement

In this study, we start by focusing on engagement, one aspect of happiness wellbeing. Of the three different forms of wellbeing, happiness wellbeing appears to be the one where researchers most often find support for the mutual gains-perspective, thus suggesting that the relationship between HPWPs and happiness is typically positive (Van De Voorde et al., 2012; Peccei and Van De Voorde, 2019).

Employee engagement describes the positive, fulfilling psychological work-related state of mind that drives employees to actively involve themselves cognitively, emotionally, and physically in performing their jobs (Schaufeli et al., 2002; Bakker et al., 2008). Existing evidence suggests that HPWPs have a positive impact on employee engagement (Bal et al., 2013; Boon and Kalshoven, 2014; Huertas-Valdivia et al., 2018; Ogbonnaya and Valizade, 2018; Alfes et al., 2021). An explanation is that HPWPs signal to the employee that the organization is invested in developing their knowledge, skills, and abilities (Gould-Williams, 2003; Sun et al., 2007), which employees reciprocate in the form of increased commitment, satisfaction, and engagement (Huertas-Valdivia et al., 2018).

The underlying theoretical framework used to explain this relationship in many of these studies is the social exchange theory (Peccei and Van De Voorde, 2019). Social exchange theory is based on reciprocity within social relationships (Blau, 1964; Emerson, 1976). The theory states that persons are motivated to repay economic, social or other benefits they receive. HPWPs such as provision of training, autonomy, team and workplace support may relay information about management’s intentions to develop a more competent and motivated workforce (Ogbonnaya and Valizade, 2018). Employees perceive these actions as a form of managerial commitment to their welfare. As a result, employees may attach a positive meaning to the intended outcomes of HPWPs and exert cognitive and physical energies at work. Based on existing research and theory, we anticipate a positive relationship between HPWPs and employee engagement.

*H1a: HPWPs are positively associated with employee engagement.*

### 2.2. High-performance work practices and workplace bullying

The second aspect of wellbeing that we examine is relationship (i.e., social) wellbeing. We address it by considering how HPWPs affect workplace bullying. Workplace bullying is defined as situations where an employee repeatedly and over a prolonged period is exposed to harassing behavior from one or more colleagues, which includes subordinates and leaders (Einarsen, 2000). Given that workplace bullying has been shown to have severe negative consequences for employee wellbeing and functioning, and for organizational performance (Nielsen and Einarsen, 2012; Hoel et al., 2020), it is important to increase our understanding of both antecedents and buffering factors. We use the absence of workplace bullying as an indication of relationship wellbeing, whilst the presence of workplace bullying is a strong indication of poor relationship wellbeing.

In the workplace bullying literature, the work environment hypothesis is used to explain how work environments can lead to incidents of workplace bullying (Einarsen, 2000; Salin and Hoel, 2020). According to Leymann (1990), stress and frustration caused by deficiencies in the workplace may lead to workplace bullying. In line with this, some researchers have feared that intensification of work and increases in stress stemming from HPWPs and high-performance expectations may increase the risk of bullying (Lewis and Rayner, 2003; Ashkanasy et al., 2016; Pichler et al., 2016). Moreover, HPWPs have been feared to increase competition among colleagues, thereby possibly resulting in more undermining and bullying (Samnani and Singh, 2014).

Although there are concerns that HPWPs may lead to bullying, there are also reasons to argue that HPWPs may in fact lead to a reduction in workplace bullying. Einarsen et al. (2019) found that high quality HR practices predicted the existence of an ethical infrastructure against workplace bullying. Moreover, HPWPs may lead to improvements in job design and in the work environment that in turn address some of the shortcomings known to be risk factors of bullying. For instance, there is overwhelming support that role conflict and role ambiguity can lead to instances of workplace bullying (for a summary, see Salin and Hoel, 2020). In line with this, Salin and Notelaers (2020) found that HPWPs led to lower role conflict, and to higher perceptions of justice, both of which in turn reduced the risk of workplace bullying.

Although arguments can be put forward both for why HPWPs would increase and why they would decrease the risk of bullying, a stronger case can be made for the latter. Based on the arguments above, we argue that:

*H1b: HPWPs are negatively associated with workplace bullying.*

### 2.3. The mediating role of organizational identification

As discussed earlier, much previous research on HPWPs and employee outcomes is based on the social exchange perspective (Peccei and Van De Voorde, 2019). However, research indicates there may also be another mechanism by which HPWPs influence employee outcomes: identification (Bartram et al., 2014; Shen and Benson, 2016; Mostafa et al., 2019). By drawing on social identity theory, we propose that organizational identification also mediates the relationship between HPWPs and employee wellbeing. Rather than employees displaying wellbeing as a result of reciprocating organizational investments in them, we suggest employees’ wellbeing could also emerge from a sense of attachment and belonging to the organization.

Defined as the “perception of oneness with or belongingness to an organization” (Mael and Ashforth, 1992, p. 104), organizational identification has its roots in social identity theory (Tajfel, 1978; Tajfel and Turner, 1985). Social identity theory posits that individuals simplify and organize the social world by a process of social categorization. They classify themselves and others into social groups such as gender, religion, ethnicity, nationality, profession and so forth. An organization may provide one such social group to which individuals link themselves. Organizational identification occurs when an employee perceives membership to the organization to be a meaningful part of his or her self-concept and perceives the fate of the organization as his or her own (Ashforth and Mael, 1989).

Social identity is most influential when individuals consider membership to a particular group to be a central part of their self-concept and develop a strong emotional tie to the group (Ashforth and Mael, 1989). A central notion in social identity theory is that individuals seek a positive social identity and thus identify most strongly with groups that enhance their self-esteem (Tajfel and Turner, 1985; Dutton et al., 1994). Organizations that appeal to employees’ self-esteem provide a relevant social group that employees readily identify with because it meets their need for self-enhancement (Dutton et al., 1994).

HR practices can influence employees’ sense of identification with the organization by meeting employees’ need for self-enhancement (Edwards, 2009; Liu et al., 2020). HPWPs communicate the organization’s investment in and commitment to its employees, and these can generate feelings of attachment and identification (Bartram et al., 2014; Liu et al., 2020). Rigorous staffing practices ensure employee-job/organization fit (Liu et al., 2020). Training and development, and rewards for good performance build employees’ self-esteem. High job involvement and employee feedback, which are often central to HPWPs, also build self-esteem as well as a sense of cohesion and shared fate with other members of the organization. HPWPs that enhance employees’ abilities, motivation, and opportunities to participate forge a positive image of the organization in employees’ minds (Appelbaum et al., 2000), which employees can incorporate into their self-concept.

In addition to appealing to employees’ self-esteem, an organization also needs to be distinctive for employees to identify strongly with it (Ashforth and Mael, 1989; Dutton et al., 1994). In other words, the organization needs to differentiate itself from others and provide an identity that is unique in relation to other comparable groups. HPWPs can enhance an organization’s distinctiveness by offering employees more competitive pay, extensive training and development opportunities, and by communicating how it values its employees (Chuang and Liao, 2010; Lee et al., 2016). Therefore, HPWPs positively influence employees’ organizational identification by appealing to their self-esteem and by differentiating the organization from other equivalent entities.

Another central idea in social identity theory is that group members seek to maintain a positive social identity by maintaining and enhancing their group’s favorable image (Ashforth and Mael, 1989). We propose that this psychological bond with the organization motivates employees to perform better and engage more with their work as they seek to maintain the organization’s positive image. We therefore hypothesize the following:

*H2a: Organizational identification mediates the relationship between HPWPs and employee engagement.*

In addition to promoting positive attitudes in employees, we propose that the effect that HPWPs have on employees’ organizational identification will result in favorable intra-group behaviors within the organization. Our proposition builds on previous empirical findings which show that organizational identification mediates the relationship between HR practices and employees’ behaviors. Shen and Benson (2016), for instance, found organizational identification mediated the relationship between certain HR practices and employees’ extra-role helping behaviors. In other words, employees who identified strongly with the organization were more likely to help their colleagues when it did not benefit themselves. Similarly, Newman et al. (2016) found evidence of organizational identification mediating the relationship between employee-oriented HR practices and employees’ organizational citizenship behaviors, which again involves ways in which employees interact with their colleagues.

Whilst previous research on HR practices and workplace bullying has principally built on the work environment hypothesis and job design, we argue that organizational identification may also act as an explanatory mechanism. HPWPs can build organizational identification by appealing to employees’ self-esteem and producing a sense of cohesion through practices such as employee voice and job involvement. Organizational identification is associated with behaviors such as cooperation, altruism, and positive evaluations of group members (Ashforth and Mael, 1989; Dukerich et al., 2002). We expect, therefore, organizational identification to result in lower occurrences of workplace bullying because other organizational members are viewed as being part of the same ‘tribe’. We hypothesize that:

*H2b: Organizational identification mediates the relationship between HPWPs and workplace bullying.*

**Figure 1:**
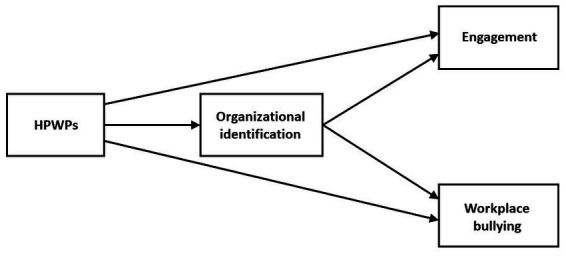
Conceptual model.

Our hypotheses can be summarized in the following diagram (Figure 1):

## 3. Materials and methods

### 3.1. Sample and procedures

The study utilized a two-wave survey design, which was chosen to reduce the risk of common method variance when testing the hypothesized model between our constructs: specifically we chose to assess the relationship between HPWP at wave 1 and the organizational identification, engagement and bullying experienced at wave 2.

The first wave questionnaire was sent out in January 2019 to all 3,937 members of the Finnish Psychological Association who had employer information registered and an email address available. Recipients received an email with a link and could fill out the questionnaire on-line in Webropol. Private practitioners were not contacted. A reminder (with a link) was sent out a month later in connection with the association’s regular newsletter. This resulted in 659 responses. The second wave survey was distributed 3 months later, and 331 people responded, with 213 of these having previously responded at wave 1. A 3 month time lag was selected in order to optimize the response rate, as we considered it possible that sending the two surveys too closely could have overloaded the respondents. The surveys and instructions were available in both Finnish and Swedish. It had been translated from English following Brislin’s (1970) recommendations concerning back-translation.

Psychologists were considered to be a particularly interesting group as much research on HPWPs so far has been from industry and business organizations. In contrast, employees in the health and social sector have received less attention and as these sectors can be seen as driven by a somewhat different logic (especially in countries where the sector is largely public rather than for profit), it offers an interesting context to further test outcomes of HPWPs.

Of the 213 respondents who took part in both waves, 193 (91%) were female. The mean age was 42.6 years (SD 11.6, range 25–66 years) - with 11.4% having worked for less than 1 year in their current organization, 20.4% for 1–2 years, 21.8% for 3–5 years, 18% for 6–10 years, and 28.4% for more than 10 years. 75% worked in the municipal sector, 11.8% in the private sector, 3.3% in the university sector and 9.9% for the state. Compared with figures for psychologists in Finland overall, psychologists from the municipal and university sectors were slightly overrepresented in our sample, although the proportions corresponded rather well to the national proportions. Women are clearly overrepresented among psychologists in Finland, which is also reflected in this study.

Whether a respondent had stayed in the study at wave 2 or dropped out after wave 1 was unrelated to any of these background variables, suggesting no systematic bias in retention. Likewise a logistic regression model, in which our key study variables (engagement, workplace bullying and HPWPs, the measurement of which is described below) at time one predicted participation/drop out in wave two, showed these variables were not statistically significantly related to participation/drop out at the *p* < 0.05 level.

### 3.2. The Finnish context

This study was conducted in Finland. Some information about the Finnish context is important to help interpret the findings. This overview focuses on aspects of the social welfare system, cultural values, and the work culture.

First of all, it is worth noting that Finland has a strong social welfare system that provides universal healthcare, comprehensive social security, and high-quality education (SSA, 2018). As for national culture, according to the seminal work by Hofstede (1980) Finnish culture is characterized by an emphasis on individualism, “feminine” or soft values, and low power distance. At the same time, the later even more comprehensive Globe study showed that Finnish culture values autonomy, independence, self-reliance, and long-term planning (House et al., 2004). High individualism, low power distance, and future-orientation may fit well with a high-performance work mindset. Interestingly, Finns value performance orientation very highly, although they get lower scores for actual performance-oriented practices. In terms of leadership, Finns value team-oriented and participative leadership styles (House et al., 2004). Overall, Finland has a work culture that emphasizes a good work-life balance, flat organizational structures, and open communication. Finland has, together with the other Nordic countries, some of the highest levels of unionization in the world. According to OECD statistics, union membership was appr. 58% in 2019, thus putting it clearly above countries such as the US and Japan and above the EU average (OECD, 2023). Overall, Finland has a strong tradition of collective bargaining and labor rights, and the high level of union membership reflects a historical commitment to promoting the interests, rights and wellbeing of workers.

All these features may thus protect Finnish workers from some of the potentially negative effects of high-performance work practices and help them leverage the positive sides in terms of both performance and wellbeing. A high emphasis on worker rights makes it difficult for Finnish employers to terminate individual employees. Due to the safety offered by strong social security, Finnish employees may also feel less compelled than employees in many other countries around the world to stay in workplaces where they feel they are badly treated or where their health and wellbeing is at risk. Furthermore, in international comparison, Finland scores high on gender equality. Finland is consistently ranked as one of the most gender-equal countries in the world (World Economic Forum, 2022). This, too, may impact the implementation of HPWPs, as well as the wellbeing of psychologists, particularly in relation to work-life balance and work–family conflict.

### 3.3. Measures

*High-performance work practices* (HPWPs) were measured with 24 items, taken from Chuang and Liao (2010). The items measured practices related to six different areas of HR: staffing (e.g., “My organization places priority on candidates’ potential to learn when recruiting employees”), training (e.g., “My organization continuously provides training programs”), participation (e.g., “Employees are often asked to participate in work-related decisions.”), performance appraisal (e.g., “Performance appraisals are based on multiple sources [self, co-workers, supervisors, customers, etc.]), compensation (“Employees receive monetary or non-monetary rewards for great effort and good performance”), and caring (e.g., “My organization has formal grievance procedures to take care of employee complaints and appeals”). Replies were given on a five-point Likert scale, ranging from 1 = strongly disagree to 5 = strongly agree.

*Organizational identification* was measured with six items (Mael and Ashforth, 1992). Sample items included “When someone criticizes my organization, it feels like a personal insult” and “When I talk about this organization,” I usually say “we rather than ‘they’.” Replies were given on a five-point Likert scale, ranging from 1 = strongly disagree to 5 = strongly agree.

*Engagement* was measured with five items from the Utrecht Work Engagement Scale (Schaufeli et al., 2006). Sample items include “At my work, I feel like I am bursting with energy” and “I am immersed in my work.” The items covered all three subdimensions of engagement: vigor (2 items), dedication (2 items) and absorption (1 item). Replies were given on seven-point, ranging from 1 = never to 7 = every day.

*Bullying behavior* was measured with the Short Negative Acts Questionnaire (Notelaers et al., 2019), encompassing nine items. Sample items included “Someone withholding information which affects your performance,” “Having insulting or offensive remarks made about your person, attitudes or your private life,” and “Being ignored or excluded.” Respondents were asked to indicate how often they had been subjected to the listed acts based on the following scale: 1 = never; 2 = now and then; 3 = monthly; 4 = weekly; 5 = daily.

#### 3.3.1. Control variables

In the study, we collected information about the following variables that we believed might confound our hypothesized relationships: gender (0 = man, 1 = woman), age (in years), and whether a person manages others (no = 0, yes = 1). Previous research has shown that both engagement and bullying may be affected by individual characteristics. In particular, some studies have suggested that men and employees in higher positions may be subjected to less bullying, whereas results have been more mixed for age (Zapf et al., 2020; Salin, 2021). As for engagement, studies have suggested that older employees and employees with supervisory responsibilities tend to report higher levels of engagement (Gostautaite and Buciuniene, 2015; Lu et al., 2016).

To test the proposed factor structure of our measures, we ran a series of Confirmatory factor Analyses using Mplus software v8. Given the small sample relative to the number of parameters, this was done in two parts. First, we assessed the wave 1 HPWP items, testing the proposed second-order structure of the Chuang and Liao (2010) items, in which 6 first order factors (corresponding to the sub-dimensions of staffing, training, participation, performance appraisal, compensation, and caring) measure a single second order overall HPWP factor. Second, we tested a 3 factor model for our wave 2 mediator and outcome scales (Organizational Identification, Engagement, Bullying), with the bullying items treated as ordinal indicators given the unequal/unquantifiable time gaps between the item response labels (e.g., 1 = never; 2 = now and then; 3 = monthly; 4 = weekly; 5 = daily).

Having removed one item with a very low communality (staffing item 4: “Qualified employees have good opportunities for promotion”, which differed from the other three items in this sub-dimension in terms of being focused on promotion rather than recruitment), the second-order 6 factor – 1 factor structure offered a good fit to the remaining 23 HPWP items (Chi-sq = 695.233 on 224 df, CFI = 0.907, RMSEA = 0.057, SRMR = 0.064). This model offered a superior fit to a simpler first-order model with 1 factor and to a simpler first-order model with 3 factors (motivation enhancing: performance appraisal and compensation items; ability enhancing: recruitment/staffing and training items; and opportunity enhancing: caring and participation items). Likewise the 3 factor model for our mediator and outcome scales offered an excellent fit (Chi-sq = 210.829 on 167 df, CFI = 0.970, RMSEA = 0.035, SRMR = 0.082), and comfortably outperformed a two factor model in which Organizational Identification and Engagement items measured a single factor. Full details of these model comparisons are given in Tables 1, 2 below.

**Table 1:** Fit of 2nd order 6 factor – 1 factor measurement model for HPWP items, and comparisons with plausible competing models.

Model	Chi-sq, df	Δ Chi-sq, Δ df	*p*	RMSEA	CFI	SRMR
2nd order 6 factor – 1 factor	695.233, 224	---	---	0.057	0.907	0.064
1st order 3 factor (motivation enhancing, ability enhancing, opportunity enhancing factors)	1694.852, 227	999.619, 3*	<0.001	0.099	0.710	0.075
1st order 1 factor	2330.068, 230	635.216, 3*	<0.001	0.118	0.585	0.088

*N* = 213, *p < 0.05. Models estimated using maximum likelihood.

**Table 2:** Fit of 3 factor measurement model for organizational identification, engagement and bullying items, and comparisons with plausible competing models.

Model	Chi-sq, df	Δ Chi-sq, Δ df^‡^	*p*	RMSEA	CFI	SRMR
3 factor	210.829, 167	---	---	0.035	0.970	0.082
2 factor (organizational identification. Engagement combined on single factor)	374.558, 169	44.248, 2*	<0.001	0.076	0.859	0.124

*N* = 213, *p < 0.05. Bullying items treated as ordinal variables, hence models estimated using weighted least squares. Model comparison therefore requires adjusted of chi-square difference test as per Muthén and Muthén (1998–2017). ^‡^Bullying items treated as ordinal indicators given the unequal/unquantifiable time gaps between the item response labels, hence model estimation by WLSMV (weighted least squares means and variances) estimator was required. Therefore adjusted chi-square difference test used to test between models, performed using the Mplus DIFFTEST procedure.

The scales defined by these four factors all had high internal consistency reliability. Specifically, at wave 1, for the 23 HPWP items, Cronbach’s alpha = 0.855; McDonald’s Omega = 0.895; at wave 2, for the organizational identification items, Cronbach’s alpha = 0.876; McDonald’s Omega = 0.920; for the engagement items, Cronbach’s alpha = 0.869; McDonald’s Omega = 0.898; for the bullying items, Cronbach’s alpha = 0.865; McDonald’s Omega = 0.917.

Our focal study constructs are all measured through self-reporting. While we acknowledge that triangulation with other methods may be advisable, we note it may be difficult to collect data on variables such as engagement, organizational identification, and experiences of bullying from other sources as they reflect highly individual experiences and the employee’s own perceptions are of crucial importance. Similarly, as HR practices are not necessarily implemented uniformly across all employees, and since employee attributions and perceptions of these differ (Nishii et al., 2008; Nishii and Wright, 2008), employee reports may again be the best option. In fact, reviews suggest that the average correlation between manager and employee perceptions of HR content is only around *r* = 0.37 (Wang et al., 2020), pointing to difficulties in using management-rated practices as outcomes are largely driven by the employee perceptions. Despite the obvious shortcomings, we therefore see employee self-reports as the most reliable option in this study.

### 3.4. Statistical analysis

To test our hypothesized model, we first computed scale mean (i.e., composite scores) across the respective sets of wave 1 HPWP items and wave 2 organizational identification, engagement and bullying items. We then fitted a path analysis model in which our mediator, organizational identification, was regressed upon our predictor HPWP and our control variables; and both outcomes, engagement and bullying, were regressed upon organizational identification, HPWP, and our controls. The outcomes were correlated. We estimated the regression coefficients for each of these paths, and then tested the total effects of HPWP onto each outcome (providing a test of H1a, H1b), and the indirect effects of HPWP onto each outcome via organizational identification (providing a test of H2a, H2b). We also added a test between the indirect paths to each outcome, to test their relative strength.

The analyses were performed using Mplus software v8, using Maximum Likelihood to fit our model. Two tailed tests, with the *p* < 0.05 level of statistical significance applied, were used throughout. Exact *p-*values (to 3 decimal places) and confidence intervals are reported throughout. When testing the indirect effects, bootstrapped 95% confidence intervals were used to assess statistical significance (Hayes, 2022).

## 4. Results

Table 3 displays the descriptive statistics and correlations of the study variables. As expected, HPWPs were positively correlated with organizational identification (*r* = 0.447) and engagement (*r* = 0.327), whereas HPWPs were negatively related with workplace bullying (*r* = −0.165).

**Table 3:** Descriptive statistics for, and correlations between the study variables.

Variable	Mean	SD	1	2	3	4	5	6
1. Sex (0 = Male, 1 = Female)	0.910	0.286						
2. Age (years)	42.624	11.600	−0.240					
3. Supervisor (1 = Yes, 0 = No)	0.038	0.191	0.062	0.128				
4. Mean scale score – HPWP, wave 1	2.683	0.520	−0.029	0.099	0.105			
5. Mean scale score – Org’ identification, wave 2	2.581	0.888	0.016	0.057	0.182	0.447		
6. Mean scale score – Engagement, wave 2	5.575	0.916	0.019	0.089	0.108	0.327	0.276	
7. Mean scale score – Bullying, wave 2	1.200	0.359	−0.059	0.000	0.035	−0.165	−0.178	−0.118

*N* = 213.

Since our variables were all observed (as opposed to latent), and all connected within the model, the hypothesized model was saturated (i.e., with a perfect fit: Chi-sq = 0 on 0 df). Table 4 displays the estimated path coefficients from our estimated model. Table 5 contains estimates of total, direct, and indirect effects upon each outcome.

**Table 4:** Estimated unstandardized path coefficients from hypothesized model.

Outcome	Organizational identification, wave 2		Engagement, wave 2		Bullying, wave 2	
Predictor	*B* (95% CI)	*p*	*B* (95% CI)	*p*	*B* (95% CI)	*p*
Sex (0 = Male, 1 = Female)	0.065 (−0.243, 0.373)	0.679	0.115 (−0.397, 0.627)	0.659	−0.084 (−0.315, 0.147)	0.473
Age (years)	0.000 (−0.010, 0.010)	0.945	0.005 (−0.005, 0.015)	0.319	0.000 (−0.004, 0.004)	0.890
Supervisor (1 = Yes, 0 = No)	0.629 (−0.188, 1.446)	0.131	0.217 (−0.418, 0.852)	0.503	0.145* (0.006, 0.284)	0.041
HPWP, wave 1	0.738* (0.542, 0.934)	<0.001	0.437* (0.176, 0.698)	0.001	−0.076 (−0.170, 0.018)	0.110
Organizational identification, wave 2	---	---	0.156* (0.025, 0.287)	0.020	−0.057 (−0.130, 0.016)	0.123

*N* = 213, *p < 0.05.

**Table 5:** Unstandardized direct, indirect and total effects of HPWPs on engagement and bullying.

Outcome	Engagement, wave 2		Bullying, wave 2	
	Estimate (95% CI^‡^)	*p*	Estimate (95% CI^‡^)	*P*
Total effect of HPWP, wave 1	0.553* (0.300, 0.806)	<0.001	−0.118* (−0.198, −0.038)	0.004
Indirect effect of HPWP, wave 1, via Organizational identification, wave 2	0.115* (0.021, 0.228)	---	−0.042 (−0.109, 0.004)	---
Direct effect of HPWP, wave 1	0.437* (0.176, 0.698)	0.001	−0.076 (−0.170, 0.018)	0.110

*N* = 213, *p < 0.05; ^‡^for direct and total effects, parametric 95% CIs are calculated. For indirect effects, bootstrapped 95% CIs are calculated, using 10,000 bootstrapped samples.

High-performance work practices had a significant positive effect on organizational identification (*B* = 0.738, *p* < 0.001), explaining 18.3% of unique variance. HPWPs had a significant positive total effect on engagement (*B* = 0.553, *p* < 0.001), explaining 11.5% of its variance, supporting hypothesis 1a. This total effect primarily operated directly (*B* = 0.437, *p* = 0.001), but there was also a significant indirect component via organizational identification (indirect effect = 0.115, bootstrapped 95% CI = 0.021, 0.228), supporting hypothesis 2a.

High-performance work practices had a significant negative total effect on engagement (*B* = −0.118, *p* = 0.004), explaining 4.4% of unique variance, and supporting hypothesis 1b. However the indirect effect on bullying via organizational identification was not statistically significant, hence hypothesis 2b was not supported.

## 5. Discussion

The study posited that HPWPs would have a positive effect on engagement and a negative effect on workplace bullying and that these relationships would be mediated through organizational identification. The results suggested that, as expected, HPWPs had a positive relationship with engagement and a negative one with workplace bullying. Furthermore, the results suggested that organizational identification acted as a mediator of the HPWP-engagement relationship, but not of the HPWP-bullying relationship.

The study makes several important contributions to the existing research. First of all, it provides additional support for the assumption that HPWPs are associated with increased, rather than decreased, employee wellbeing. In the HPWPs literature there is a longstanding debate on whether HPWPs produce better performance at the expense of employee wellbeing or rather through improved employee wellbeing (Peccei and Van De Voorde, 2019). Our study provides support for the latter alternative by reporting both higher engagement and less bullying. Moreover, so far there has already been rather extensive research on HPWPs and different aspects of the happiness aspects of wellbeing (Van De Voorde et al., 2012; Peccei and Van De Voorde, 2019). In contrast, our understanding of the relationship between HPWPs and relationship wellbeing is still much more limited. By finding a negative relationship between HPWPs and workplace bullying our study thus contributes to this rather scant research, suggesting that HPWPs improve also relationship wellbeing.

Secondly, our study increases our understanding of the mechanisms linking HPWPs and employee outcomes. Whereas previous research has largely relied on a social exchange perspective (Peccei and Van De Voorde, 2019), our study provides an alternative point of view by drawing upon social identity theory and presenting organizational identification as an alternative mediator. Our results support the assumption that organizational identification acts as a partial mediator between HPWPs and engagement. This suggest that to some extent the effect of HPWPs on employee wellbeing could stem from a sense of attachment to and belonging to the organization. It suggests HPWPs can influence employees to see the organization as a meaningful part of their self-concept and help them meet their need for self-enhancement (*cf.* Liu et al., 2020).

Although our study found organizational identification to be a mediator of the HPWPs-engagement relationship, the same was not found for the HPWPs-bullying relationship. There are several possible explanations for this. First, our study looked at exposure to bullying rather than perpetration of bullying. It is possible that organizational identification will reduce the employee’s own tendency to engage in negative acts toward colleagues, but that it cannot necessarily protect against negative acts from others. Second, bullying scores were overall exceptionally low in this sample, suggesting bullying may not have been a large problem in this group to start with. Even so, in line with some previous empirical research (Salin and Notelaers, 2020; Salin et al., 2022) this study demonstrated a negative rather than positive relationship between HPWPs and workplace bullying. This thus contradicts prevailing concerns that HPWPs may rather increase than decrease the risk of bullying and other forms of employee mistreatment (Lewis and Rayner, 2003; Samnani and Singh, 2014; Ashkanasy et al., 2016).

### 5.1. Practical implications

Overall, the results of this study highlight the positive effects of HPWPs. HPWPs result in higher organizational identification, which has many positive effects on employee wellbeing and behavior (Greco et al., 2022), among them higher engagement as demonstrated in this study. By investing in different aspects of HPWPs, such as advanced recruitment and selection methods, extensive training, employee participation and involvement, rigorous performance appraisal, competitive compensation and HR practices that demonstrate care for employee wellbeing (i.e., the practices included in this study), organizations may foster a sense of attachment and belonging to the organization, This in turn translates into higher engagement, which in itself means higher levels of employee wellbeing, but also has been linked to better performance (Alfes et al., 2013a,b).

Furthermore, the results suggest that HPWPs decrease the risk of bullying, Given the high costs associated with workplace bullying, both in terms of decreased employee health and wellbeing and costs associated with absenteeism, turnover and lower productivity (e.g., Nielsen and Einarsen, 2012; Hoel et al., 2020), this is of utmost importance. Although the effects of HPWPs have been highly contested (*cf.* Samnani and Singh, 2014; Ashkanasy et al., 2016), this study provides additional support for the view that HPWPs improve rather than worsen the work environment and thereby reduce the risk of bullying (*cf.* Salin and Notelaers, 2020). In addition to typical remedies to reduce bullying, such as having a zero-tolerance policy and providing training (Salin, 2020), this study points to the importance of high-quality HR practices as a means of reducing the risk.

### 5.2. Limitations and future research

This study relied on self-report data. There is thus a risk of common-method variance bias (Podsakoff et al., 2003). Nevertheless, given the highly subjective nature of the focal variables, we consider employee self-reports as the most reliable option in this study, as discussed in more detail in the section about measures. Different strategies can be employed in order to try to diminish common method variance. Podsakoff et al. (2003) have suggested different possible remedies. In this study, the independent variable and the dependent variable were collected at different points in time, with an approximately 3 month time lag. Such a temporal separation through a time lag is one way of reducing the risk of common method variance bias (Podsakoff et al., 2003). Other possible remedies include ensuring anonymity and thereby reducing responder apprehension and ensuring item clarity to facilitate comprehension and avoid ambiguity (Podsakoff et al., 2003). When designing our survey, we paid close attention to these recommendations.

In this study, we did not control for engagement or bullying at time one. Then again, we have no reason to expect a change in these variables between the two time points. It is most likely that the benefits accrued from the HPWPs in place have accrued over a longer period of time, and thus any change during this specific time frame, as there has been no change in HPWPs, would be unlikely. This is in line with the findings by Guest et al. (2003) in their study on HRM practices and corporate performance.

In this study, we focused on engagement and bullying, that is, aspects of happiness and relationship (social) wellbeing. According to Grant et al. (2007), health is typically acknowledged as the third form of wellbeing. However, we had no measure of health in our study. While we theoretically could identify arguments for why organizational identification would be likely to mediate the relationship between HPWPs on the one hand and happiness and social wellbeing on the other, we had no clear rationales for why this would be the case for health. Still, analyzing also the mediating effect for a health-based outcome could be a suggestion for further research.

Moreover, our study was undertaken within one specific sector, that is among psychologists. This sector is heavily female-dominated, at least in Finland. Also, much of the work is done with individual clients and collaboration between colleagues may be lower than in many other occupations. Furthermore, the sample size was relatively small, given the relatively high drop-out rate between wave 1 and wave 2. All this places limits on to what extent we can generalize the findings. Moreover, given our interest in mediation, ideally, we would have preferred to collect data at three time points: first the independent variable (HPWPs), then the mediating variable (organizational identification), and finally the outcome variables (engagement and bullying). However, for practical reasons we had to restrict data collection to two time points. We therefore strongly encourage researchers to try to replicate our study with a three-wave design and to also study other sectors, occupations, and cultural contexts.

Furthermore, recent research has called for more research that analyze the interacting effects of HR and leadership (Leroy et al., 2018). So far, these two concepts have typically been studied in isolation, although emerging findings suggest their joint influence should be acknowledged and examined (McClean and Collins, 2019; Hai et al., 2020; Hauff et al., 2022; Salin et al., 2022). For future research, we therefore recommend also studying the effects of leadership on these relationships. For instance, moderated mediation could be used to establish whether either a positive form of leadership such as transformational leadership, or a negative form of leadership such as *laissez-faire* leadership would further enhance or decrease the relationships examined in our study.

## 6. Conclusion

The study drew upon social identity theory and set out to study how high-performance work practices affect engagement and workplace bullying, and to examine if organizational identification acts as a mediator in these relationships. Data were collected among psychologists in Finland. The results partially supported the hypotheses. The results showed that high-performance work practices (HPWPs) were positively associated with engagement and negatively associated with the risk of workplace bullying. Organizational identification acted as mediator of the HPWPs-engagement relationship, though alongside the significant indirect effect via organizational identification there was also a significant direct effect of HPWP on engagement. However, organizational identification did not mediate the HPWPs-bullying relationship. Overall, the results point to the importance of high-performance work practices in creating a healthy work environment, with higher organizational identification, higher engagement, and less bullying.

## Data availability statement

The raw data supporting the conclusions of this article will be made available by the authors, without undue reservation.

## Ethics statement

Ethical review and approval was not required for the study on human participants in accordance with the local legislation and institutional requirements. Written informed consent for participation was not required for this study in accordance with the national legislation and the institutional requirements.

## Author contributions

DS was the lead author and took main responsibility for developing the research idea and conceptualization, designed the questionnaire, oversaw the data collection, and wrote most parts of the manuscript. CS was responsible for the statistical analyses and for writing up the results of the analyses. SoS generated the original idea together with DS, provided input on data collection, and wrote the sections about organizational identification. StS conducted a literature search, helped with writing up the theoretical framework, and conducted preliminary analyses for a conference presentation. All authors commented on previous versions of the manuscript and all approved the submitted version.

## Funding

This work was supported by the Academy of Finland under Grant 308843. Also, SoS received funding from the Ella and Georg Ehrnrooth Foundation.

## Conflict of interest

The authors declare that the research was conducted in the absence of any commercial or financial relationships that could be construed as a potential conflict of interest.

## Publisher’s note

All claims expressed in this article are solely those of the authors and do not necessarily represent those of their affiliated organizations, or those of the publisher, the editors and the reviewers. Any product that may be evaluated in this article, or claim that may be made by its manufacturer, is not guaranteed or endorsed by the publisher.
